# A Flash Group Creation Algorithm for P300 Brain–Computer Interface Integration with Irregular Assistive Technology Keyboard Layouts

**DOI:** 10.3390/s26072123

**Published:** 2026-03-29

**Authors:** Jane E. Huggins, Palash Biswas, James K. Huggins, Rishabh Chandel

**Affiliations:** 1 Department of Physical Medicine and Rehabilitation, University of Michigan Medical School, Ann Arbor, MI 48109, USA; 2 Department of Biomedical Engineering, College of Engineering, University of Michigan, Ann Arbor, MI 48109, USA; 3 Department of Statistics, College of Literature, Science, and the Arts, University of Michigan, Ann Arbor, MI 48109, USA; 4 Department of Computer Science, Kettering University, Flint, MI 48504, USA; jhuggins@kettering.edu; 5 Department of Electrical Engineering and Computer Science, College of Engineering, University of Michigan, Ann Arbor, MI 48109, USA; rchandel@umich.edu

**Keywords:** human–computer interface, augmentative and alternative communication, event-related potentials

## Abstract

An event-related potential (ERP)-based brain–computer interface (BCI), or P300 BCI, has long been intended for communication access for individuals with severe motor impairments. BCI access to communication tools, websites, and augmentative and alternative communication (AAC) keyboards requires aligning BCI stimuli to screens with differing numbers of various-sized keys in partially populated grid layouts. Six design priorities were defined for creating and ordering flash groups: identifiability, unpredictability, perceptibility, minimality, anti-adjacency, and equality. Building on the checkerboard paradigm, multiple algorithmic approaches were evaluated on simulated AAC screens to create the magic square paradigm (MSP) for flash group creation for irregular key layouts. The MSP algorithm was then used for BCI access to the dynamic screens of a commercial AAC device that combines text-based and icon-based language representations and the resulting flash groups analyzed for design priorities of anti-adjacency and equality. The 126,944 flash groups created for 5778 selections on AAC screens had 0 groups with side-by-side adjacency, 0.02% with adjacency to an amalgamated key, and 6% with diagonally adjacent keys. The average difference between the shortest and longest flash groups was 1.9 keys. The MSP provides a novel method to access dynamic AAC keyboards with irregular layouts and multiple key sizes.

## 1. Introduction

Brain–computer interfaces (BCIs) interpret brain activity to allow users to operate an external device without muscular control. Such interfaces can, in principle, provide alternative methods to communicate, control devices, and interact with the surroundings for people with severe physical impairments. One of the earliest and most effective non-invasive BCI designs for communication is the event-related potential (ERP)-BCI, introduced by Farwell and Donchin in 1988 [1] and also known as the P300 BCI design or P300 speller. The basic mode of operation of an ERP-BCI has not changed much since the first proof-of-concept device more than thirty years ago. The established paradigm of key selection in an ERP-BCI involves a sequence of stimulus presentations. Groups of keys in an on-screen keyboard are presented to the user and the brain activity in response to this stimulus is recorded as electroencephalogram (EEG) signals. Using a binary classifier previously calibrated for the specific user, the EEG response to each stimulus is classified as target ERP present or target ERP absent. Consecutive sequences of stimulus presentations and detection of ERP responses enable identification of the user’s intended key. In subsequent years, improvements in stimulus design, the addition of word prediction features, or new applications have produced ERP-BCIs that are usable for communication [2] or recreation [3] in long-term independent use in patients with severe muscular disorders.

### 1.1. Evolution of BCI Design

Advances in BCI design have largely focused on the stimulus presentation methods. In contrast to the original ERP-BCI where gray keys on a black background change to white, stimuli have become richer and more complex. Changes to stimuli like presentation of multiple colors [4,5,6] or images such as faces, objects, and icons as stimuli have improved BCI performance [7,8,9]. Stimuli in the form of human faces can improve BCI performance by eliciting additional ERP components [10,11,12,13].

The design of ERP-BCIs is a good fit for keyboard-based applications since the design relies on the user’s binary response (ERP present or not) to the presentation of stimuli. A “keyboard”-based application does not necessarily mean a letter-based application, since the keys could contain any type of content. Instead, a keyboard should be considered a two-dimensional menu of options that the user can access as desired. Besides communication, there has been a steady effort to expand the usage of P300 ERP-BCIs to other functions, including web surfing [14,15,16], driving a wheelchair [17], creating digital artworks [18], music composition [19], and gaming [20]. These diverse applications have expanded the uses of ERP-BCIs but still rely on a static keyboard of options for the user to select from, whether letters, musical notes, colors, or directions. Further, the creation of purpose-built BCIs limits available applications to those that BCI developers have worked on. A better approach is to treat BCIs as interfaces to existing assistive technologies. While standalone BCI systems progress one at a time, that approach cannot compete with the range and flexibility of language features provided by augmentative and alternative communication (AAC) technology developed over the past fifty years. BCIs as the access method to AAC devices will better satisfy communication needs.

Only a few studies have examined integrating ERP-BCIs to operate existing assistive technologies or other software. BCI use with commercial word prediction applications retained the static BCI keyboard [21,22]. Integration of an ERP-BCI into a comprehensive assistive technology suite included either a static keyboard or flashes of individual keys [23]. In stark contrast to the fixed, fully populated grid used by most ERP-BCIs, general-use software involves complex user interfaces with multiple selectable items of differing sizes distributed over the screen in a non-contiguous fashion. Thus, extending BCI access to operate the wide variety of general-use programs that have become an essential part of daily life requires a new paradigm.

Our work is intended to provide ERP-BCI access to a commercial line of AAC devices. In the process, we develop broader design guidelines that can be constructive for future works in integrating ERP-BCI access to either AAC devices or devices intended for other uses.

### 1.2. AAC System Design

**Figure 1:**
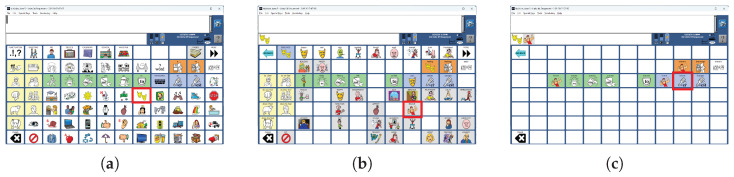
Navigating semantic compaction and selecting words on a MINSPEAK^™^ keyboard [24]. The AAC keyboards are dynamic as the number and icons of the keyboard can change after every click. (**a**) Selecting the key ’Feel’ (highlighted in red border, row 4, column 8); (**b**) subsequently selecting the key ’Brave’ (highlighted in red border, row 5, column 9); (**c**) finally selecting the word ’Braver’ (highlighted in red border, row 3, column 10).

Our work focuses on BCI access to an AAC product line (PRC-Saltillo, Wooster, OH, USA). The product line offers multiple language representations presented on a dynamic keyboard. Screens can be developed with unique layouts to meet the needs of individual users. Screens can also grow with the user as they learn more complex language concepts. Users can transition between language representation methods to improve communication speed. The Unity language representation combines a letter-based keyboard, word prediction, and an icon-based language representation called MINSPEAK^™^ [24]. MINSPEAK^™^ provides efficient communication through icon sequences instead of letter-by-letter spelling. MINSPEAK^™^ uses ‘semantic compaction’ to group related words into icon sequences starting with the same icon. On the icon-based keyboard, the user does not spell out each character of a word, but instead selects the icon sequence for the word. Once the first icon is selected, the keyboard changes to remove icons that would not be part of a valid icon sequence. For example in Figure 1, producing the word ‘Braver’ requires the user to first select the key ‘Feel’ (row 4, column 8). The keyboard then changes to show various words related to ’Feel’. The user then selects the key for ‘Brave’ (row 5, column 9) and finally the key for ’Braver’ (row 3, column 3).

The user can select precisely the word they want by navigating to a sufficient depth in the icon sequences. The paradigm has been shown to reduce the number of choices required to spell out each word, increasing communication rates by 282% [25]. For ERP-BCI access, the reduced number of keys at deeper levels of the tree (Figure 1) has the potential to speed communication rates by requiring less time to present all stimuli. Such semantic-tree based keyboards are different from conventional BCI keyboards in three ways. First, the different levels of the semantic tree have different numbers of keys. Second, a single keyboard can have keys of different sizes and orientations. Third, these keys might be arranged in any configuration on the screen and not necessarily in a neat matrix. The potential combinations of key numbers, shapes, orientations and arrangements are a large enough space to rule out any pre-programmed stimulus pattern for each keyboard.

### 1.3. Design of Flash Groups

Since the introduction of the ERP-BCI, the BCI keys have been presented in flash groups (stimulus groups). A *flash group* is a group of keys that are all highlighted at the same time. The purpose of the stimulus presentation is to elicit a strong ERP response to the group containing the target key while minimizing the response to groups that do not contain the target key. The preferred response has a large amplitude and consistent latency. The algorithm by which the flash groups are created can affect these ERP characteristics.

**Figure 2:**
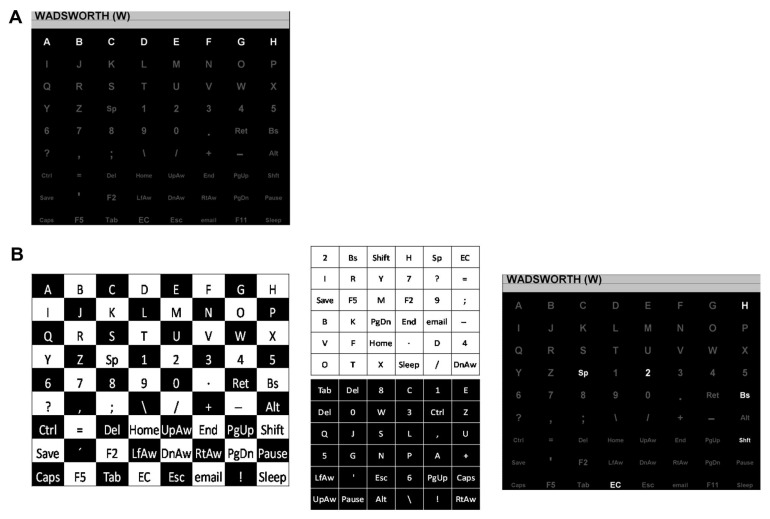
Reprinted with permission from [27]. (**A**) The row–column paradigm (RCP) for the 8 × 9 matrix, with one row flashing. (**B**) The checkerboard paradigm (CBP) for the 8 × 9 matrix. On the left is the checkerboard pattern. In the middle are the two virtual 6 × 6 matrices derived from the checkerboard. On the right is the matrix as presented to the participant with the top row of the white 6 × 6 virtual matrix flashing.

The original Farwell and Donchin ERP-BCI [1] used what is now referred to as the row–column paradigm (RCP) to create flash groups (Figure 2a). The user is presented with a matrix of characters (grey on a black background). The rows and columns of the matrix light up (change color from grey to white). Thus, the rows and columns are the flash groups, and the ERP response to each flash group is recorded. When the user focuses attention on a specific key on the grid, the flashes of the row and the column that contain that key will elicit an ERP response, while the rest of the rows and columns will not. This setup of the matrix allows the BCI to uniquely determine the user’s intended key. Once the row and the column that produce an ERP are identified, the target key is at the intersection of that row and column. By shuffling the order in which the rows and columns flash, the novelty of the target stimulus is maintained. The RCP paradigm has been considered the default flash group creation algorithm, since the majority of BCI research is bench-marked against it.

The flash groups are flashed in a pseudo-random pattern, with the condition that all flash groups must be presented once before any are repeated. Thus, a *sequence* of stimuli is defined as one presentation of every flash group and includes two presentations of each key. In the original Farwell and Donchin ERP-BCI, this meant flashing each row and column of the grid exactly once. Theoretically this means that a key could be detected from the responses for one sequence, since that involves presentation of all the flash groups and detecting the ERP response in one row and one column would identify the key. However, EEG has a low signal-to-noise ratio (SNR) [26]. So in most BCIs, multiple sequences of stimuli are presented for one selection. While multiple sequences generally increase the accuracy of key selection, they also increase the time taken to select one character.

The earliest efforts to move beyond the row-and-column style of stimulus presentation were made by Townsend et al. [27]. The work created what was called the checkerboard paradigm (CBP) for flash groups (Figure 2b). The first CBP implementation was on an 8 × 9 keyboard. The 72-key rectangular keyboard is overlaid with a hypothetical black-and-white checkerboard. The keys are separated into two groups based on the color of the checkerboard location they fall in (set of black keys and set of white keys). The black keys and the white keys are arranged in two 6 × 6 matrices, called the *virtual matrices*, which are hidden from the user. The rows and columns of the two virtual matrices form the flash groups. On the actual keyboard visible to the user, these keys are scattered across the screen. The BCI user remains oblivious to the checkerboard split and the arrangement of keys in the virtual matrices, thus maintaining the novelty of the stimuli. Since the rows and columns of the virtual matrices are the flash groups, the CBP can uniquely determine each key by identifying two target groups. The checkerboard split ensures that all keys that are side-adjacent on the actual keyboard are part of different virtual matrices. Since a single stimulus can only contain keys from the same virtual matrix, the CBP can ensure that no adjacent keys flash simultaneously.

The CBP has another advantage. During the stimulus presentation, the virtual rows of the first virtual matrix are presented in a randomized order, followed by the virtual rows of the second virtual matrix (in random order). The virtual columns of the first matrix are presented next, followed by the virtual columns of the second virtual matrix. This order imposes a minimum number of intervening flashes before a key is flashed a second time. This increase in the target-to-target interval (TTI)—the time duration between two flashes of the same item—eliminates problems caused by double flashes [27] and increases the P300 amplitude [28]. Double flashing occurs when the same key flashes in consecutive flash groups; because successive flash groups are presented rapidly, double flashing makes it difficult for the user to perceive and respond to the second target flash. Enforcing a minimum target-to-target interval also decreases the possible amount of overlap in the EEG segments following flashes of the same target.

When considering a flash group creation algorithm to apply a screen that could include keys of multiple sizes or shapes that may be sparsely distributed across the screen, we find that both RCP and CBP algorithms have shortcomings. RCP flash groups are inappropriate for a screen with a sparse arrangement of keys since some rows may have only a few keys, or none at all, while others may contain many keys. Likewise, CBP flash groups are inappropriate for a screen with multiple key sizes since the checkerboard by itself is not sufficient to prevent adjacent keys from flashing together.

## 2. Methods

Section 2.1 defines design priorities for creating BCI flash groups to access other software. Section 2.2 describes the unique characteristics of the AAC screens that inspired specific elements of the new algorithm. Section 2.3 gives an overview of the algorithm and its primary design parameters. The specifics of the parameter selected for each design decision are described in detail; the other options evaluated are described in Appendix A. Section 2.4 describes the offline analysis and simulations used to select the parameters. Section 2.5 describes design decisions made during implementation for online use. Finally, Section 2.6 describes analysis of algorithm performance data available from online use.

### 2.1. Design Priorities

Drawing on the principles established for ERP-BCI detection by the RCP and CBP flash group algorithms, we define six design priorities to guide our choices for the flash group creation algorithm. The guidelines help in efficient stimulus presentation. If tradeoffs are necessary, then the design priority with the smaller rank takes precedence.

- In an ERP-BCI, any target key (user-intended key) must be identifiable from a series of binary signals (of whether a P300 ERP is present or not). The binary series of responses to a sequence of stimuli must be able to identify every key of the keyboard uniquely. All keys of the keyboard must be interpreted (identified) from their unique binary sequence. This gives us the first and foremost design priority, *identifiability* **(DP1: Identifiability)**.
- The P300 ERP, critical to BCI operation, is generated in response to a stimulus that is anticipated by the user but unpredictable [28]. The flash groups generated by the algorithm must not be easily predictable. The second design priority for the algorithm is *unpredictability* **(DP2: Unpredictability)**.
- The users’ ERP responses to stimuli occur over a finite time and ERP-BCIs often analyze the EEG response in the 800 milliseconds (ms) after the presentation of the stimuli (e.g., [11,22]). If the stimulus presentation is so rapid that consecutive flashes of the same stimuli occur in a time shorter than this interval, the latter stimuli might be missed by the user. Further, ERP responses to rapid stimuli have greater temporal overlaps, which results in an inconsistent EEG waveform and amplitude [8]. To overcome this, flash groups must be presented in a way that ensures that the target-to-target interval of any key is long enough that both stimuli are perceptible. The third design priority is *perceptibility* **(DP3: Perceptibility)**.
- A brute-force method for stimulus presentation is to have each key on the screen presented separately. While such a solution satisfies DP1 through DP3, as the size of keyboards grow, it takes increasingly longer times for stimulus presentation. To enhance the speed of the BCI, keys must be presented in groups and the number of such groups must be as few as possible. This gives us the fourth design priority: *minimality* **(DP4: Minimality)**.
- Fazel-Rezai (2007) presents evidence that locations adjacent to the target key [29] are responsible for the majority of misclassification errors. To reduce the errors, the adjacent keys must not be part of the same stimulus. The design priority capturing this idea is *anti-adjacency* **(DP5: Anti-adjacency)**.
- A deliberate effort is made to keep the stimuli consistent in size and appearance. As a principle, the size of all flash groups is roughly equal for a given keyboard. The final guideline is *equality* **(DP6: Equality)**.

Using these design principles as guidelines, we create and evaluate a novel flash group creation algorithm to convert any arbitrary configuration of keys on a rectangular grid into a set of *flash groups*: groups of keys that are presented as stimuli simultaneously. The new algorithm retains certain components of the CBP, including the virtual matrices and the order in which the flash groups are presented (randomized virtual rows followed by randomized virtual columns). The algorithm takes into account the location, shape, size, and configuration of the keys of the on-screen keyboard and optimizes each of the design priorities. Multiple options were evaluated for the algorithmic elements of dividing the keys between the virtual matrices, selecting the size of the virtual matrices, selecting the order of key placement within the virtual matrices, and arranging the keys within the virtual matrices. These options were evaluated to determine their effect on the design priorities. Further, the algorithm includes a randomization element by which different flash group sets can be created from the same keyboard. We tested the final algorithm over a variety of keyboards, real and simulated, to evaluate the algorithm’s performance in meeting each of the design priorities.

### 2.2. Interface Specifications

**Figure 3:**
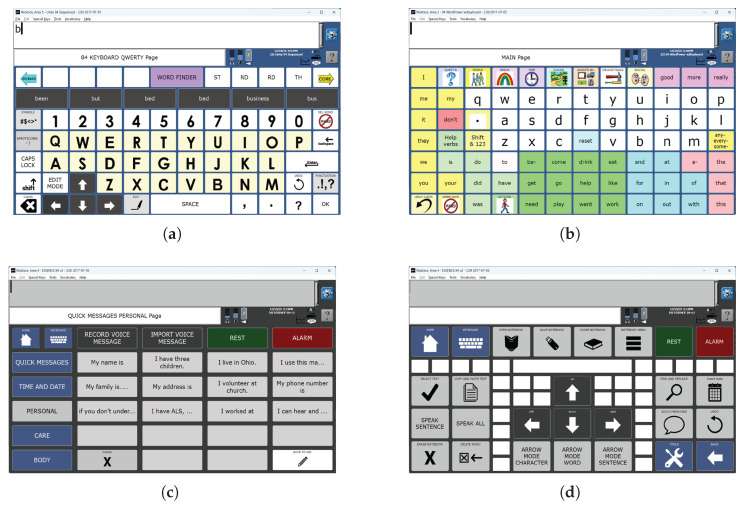
Letter-based (**a**), word-based (**b**), and stored message (**c**,**d**) AAC keyboards. Note that keys can occupy more than one grid location and some grid locations may not contain an active key.

This flash group creation algorithm was designed to create sets of flash groups (stimulus groups) for BCI access to the language representations used by commercial AAC devices from PRC-Saltillo, Wooster, OH, USA. The algorithm was therefore tested only on AAC device keyboards (collectively referred to as *AAC keyboards*) that can support the multiple language representations available on these devices. These language representations include icon-based (Figure 1), letter-based (Figure 3a), and word-based keyboards (Figure 3b) as well as stored messages (Figure 3c,d).

**Figure 4:**
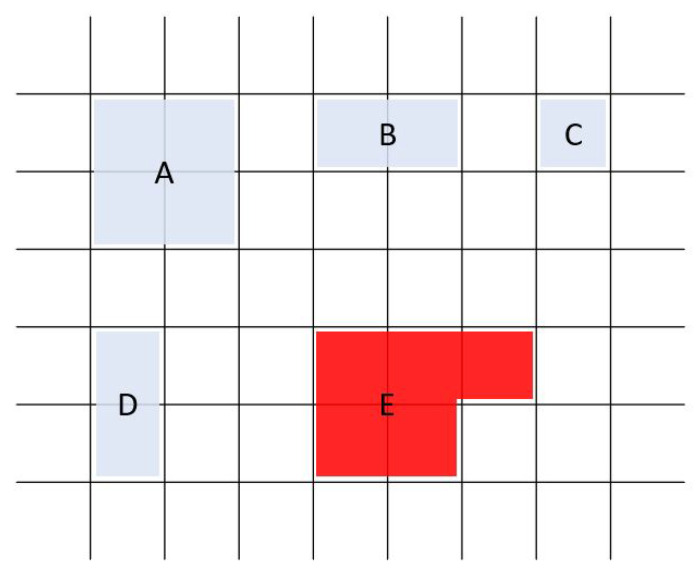
Rectangular grid and key shapes. Keys in AAC keyboards are based on blocks of an underlying rectangular grid. While keys may contain just one block (key C) or multiple blocks (amalgamated keys A, B, and D), they must be strictly rectangular in shape. An incomplete rectangle (key E) is not part of these AAC keyboards and the algorithm has not been tested for such keys.

The AAC keyboards are based on a rectangular grid so that each key is defined by its position on the grid. The maximum grid size is 9 × 16 (144 grid locations). The grid also provides an objective numerical definition of adjacency. Some AAC keyboards include keys that are composed of multiple grid locations, which we call amalgamated keys (Figure 3a,c,d and Figure 4). Keys are not always present in every grid location; that is, a keyboard may have ‘holes’ (Figure 3a,d). We refer to these grid locations as inactive in contrast to the grid locations with an active key that can be clicked (or selected). Thus, a keyboard may have fewer keys than the total number of available locations on its underlying grid.

For our algorithm, we define two properties of the keys.

**Figure 5:**
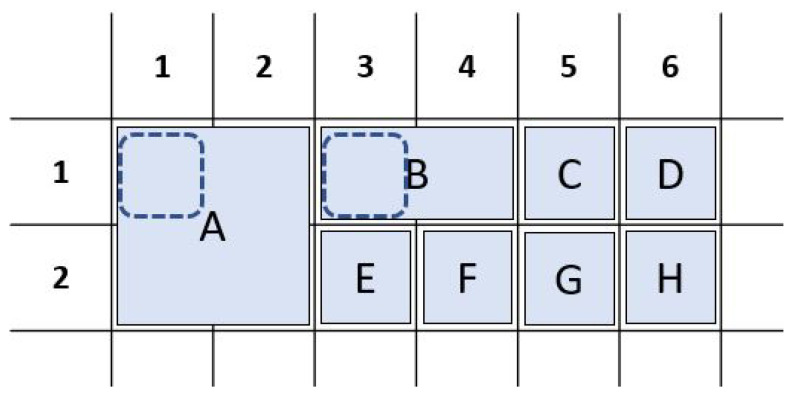
All keys are said to correspond to the topmost and the leftmost grid locations they occupy. Amalgamated keys A and B correspond to the grid locations row 1, column 1 and row 1, column 3 respectively. Keys C through H correspond to the only grid location they occupy.

**Figure 6:**
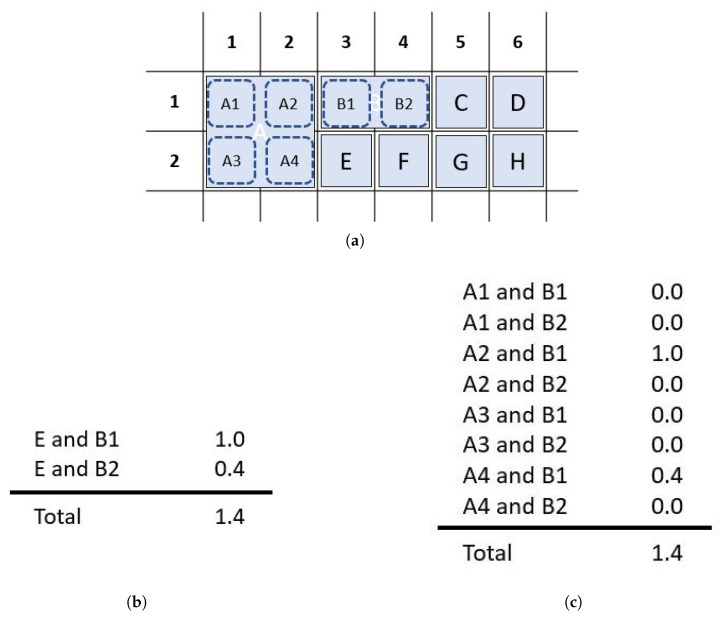
Adjacency calculations that include amalgamated keys (i.e., keys that occupy multiple grid locations): The adjacency between any two keys is the sum of the pairwise adjacencies of the grid locations occupied by the keys. (**a**) Sample keyboard with two amalgamated (multi-cell) keys. Key A occupies 4 grid locations and key B occupies 2 grid locations. (**b**) Adjacency between amalgamated key B and single-cell key E. (**c**) Adjacency between amalgamated keys A and B.

- Key-to-grid correspondence: In a traditional BCI keyboard, a key is uniquely determined by the row and the column it occupies on the grid. In the AAC keyboards, amalgamated keys span multiple grid locations. Since the AAC keyboards only contain rectangular keys, every key can be uniquely determined by its top-left and bottom-right grid locations. By convention, we use the top-left key number as the identifier for the key (Figure 5).
- Adjacency: In traditional BCI keyboards, two keys are adjacent if they are next to each other in the same column or row. Two keys that share a corner can be called diagonally adjacent. In AAC keyboards, where keys are not just restricted to a single row and a single column, we extend the concept of adjacency as a numeric metric. There are two basic rules for calculating the adjacency between two grid locations.
  – The adjacency between two grid locations is 1 if they immediately neighbor each other on the same row or the same column of the rectangular grid (side adjacency).
  – The adjacency between two grid locations is 0.4 if they share a corner of the rectangular grid (diagonal adjacency).
  The chosen adjacency values create an algorithmic preference for diagonal adjacency over side adjacency (row or column). The value of 0.4 was chosen empirically based on the observation that a value of 0.5 resulted in occasional key placements with row and column adjacency. No other values were evaluated. With a value of 0.4 for diagonal adjacency, key placement diagonally adjacent to two other keys is preferable to a side-adjacent key placement in an adjacent row or column.
  The *amalgamated adjacency* between two keys on an AAC board is the sum of the adjacencies between pairs of the grid locations, one from each key (Figure 6). It should be noted that this definition of the metric implies that adjacency is symmetric. Further, adjacency provides a numeric objective to minimize as per design priorities (DP5: Anti-adjacency).

Extension of the flash group creation algorithm to screens without an underlying grid structure or with non-rectangular keys should be possible, but is outside the scope of this work. Extension of the algorithm to an arbitrary layout of keys could be accomplished by adding an algorithm to derive a grid structure compatible with the layout. Extending to non-rectangular keys would require changes to the method of specifying key sizes.

### 2.3. Algorithm Overview

**Figure 7:**
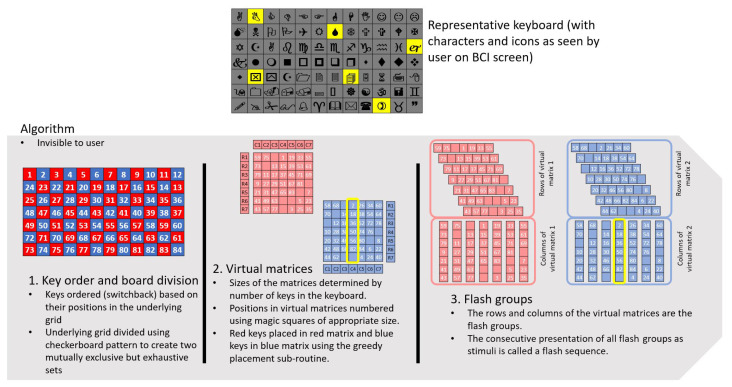
Overview of the magic square paradigm (MSP) with column 4 of the blue matrix flashing. The keys on the board are ordered in a switchback manner and divided into two mutually exclusive groups. The keys are placed into two square virtual matrices (possibly of different sizes) using a variation on a magic square placement algorithm. The rows and columns of the virtual matrices are the flash groups. The order of presentation within the virtual rows and columns is varied for each sequence.

**Figure 8:**
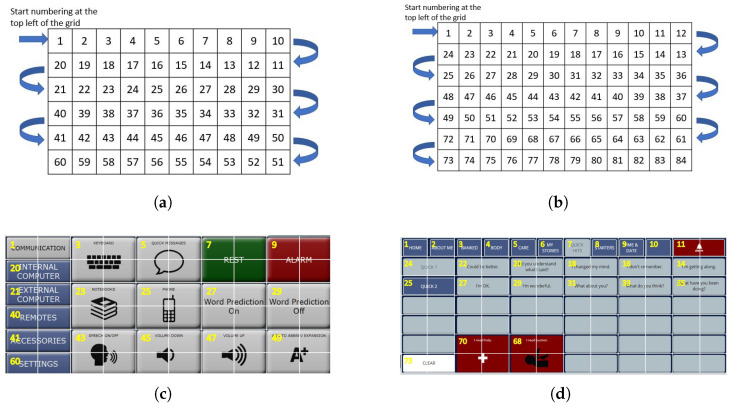
Illustrations of switchback key order. Notice that while all grid positions are numbered, the ordering of a key is based only on the number of the top-left grid cell it occupies. Inactive key locations are not assigned a number. (**a**) Switchback order for 6 × 10 grid. (**b**) Switchback order for 7 × 12 grid. (**c**) Switchback ordering of a keyboard based on the 6 × 10 grid. (**d**) Switchback ordering of a keyboard based on the 7 × 12 grid.

**Figure 9:**
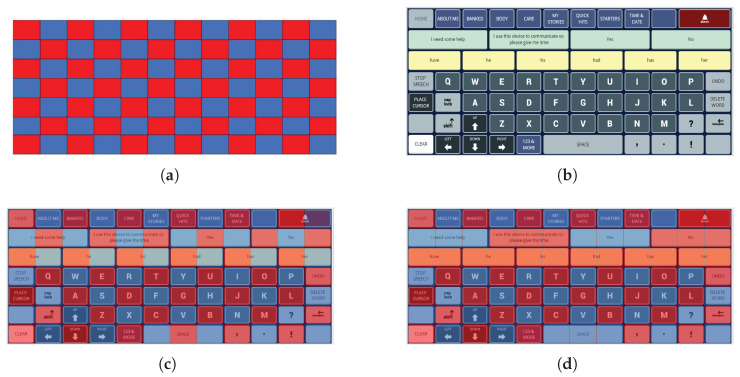
Illustration of board division. The keys are allotted to the red and blue virtual matrices based on what color aligns with the topmost and leftmost grid location of the key. In this example, the key ‘I need some help’ (row 2, columns 1 through 3) is assigned to the blue virtual matrix. The key with the bell icon (row 1, columns 11 and 12) is assigned to the red virtual matrix. (**a**) Red–blue checkerboard for an 84-location (7 × 12) grid. (**b**) Sample AAC keyboard based on an 84-location grid. (**c**) Overlaying checkerboard pattern on a sample keyboard (**d**) Expanded overlaying checkerboard pattern shows matrix assignments.

**Figure 10:**
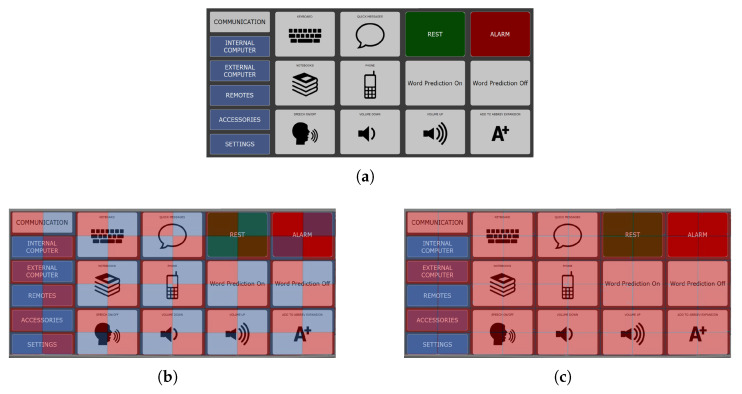
Illustration of the over-capacity matrix. The AAC keyboard (**a**) has only 18 keys, and hence the virtual matrices are of sizes 3 × 3 each (each can accommodate 9 keys). However, the many merged keys with the upper corner under a red checkerboard square (**b**) cause only three keys (**c**) to be allotted to the blue matrix (‘Internal Computer’ at row 2, columns 1 and 2, ‘Remotes’ at row 4, columns 1 and 2, and ‘Settings’ at row 6, columns 1 and 2. The rest of the 15 keys are assigned to the red matrix, which can only accommodate 9 of them.

**Figure 11:**
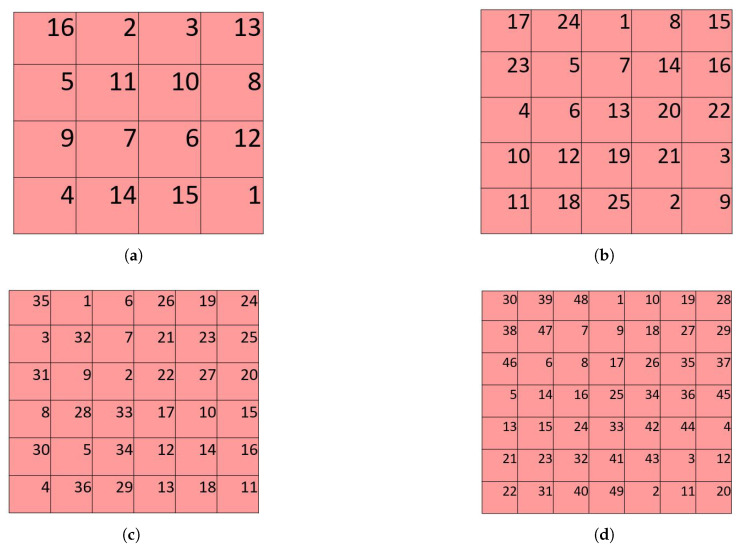
The magic squares used in the algorithm. The numbers of the cells (in superscript) indicate the order in which the cells of the virtual matrices are filled. (**a**) 4 × 4 magic square. (**b**) 5 × 5 magic square. (**c**) 6 × 6 magic square. (**d**) 7 × 7 magic square.

**Figure 12:**
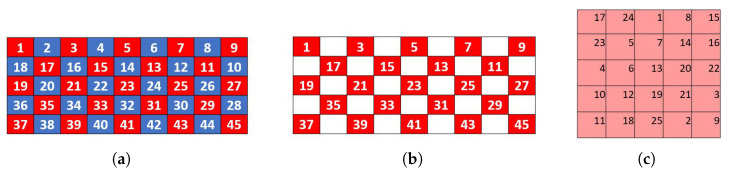
Inputs to the anti-adjacency placement algorithm. (**a**) Key order and red–blue checkerboard for a 45-location (5 × 9) grid. (**b**) The 23 keys allotted to the red 5 × 5 virtual matrix. (**c**) The 5 × 5 magic square virtual matrix.

**Figure 13:**
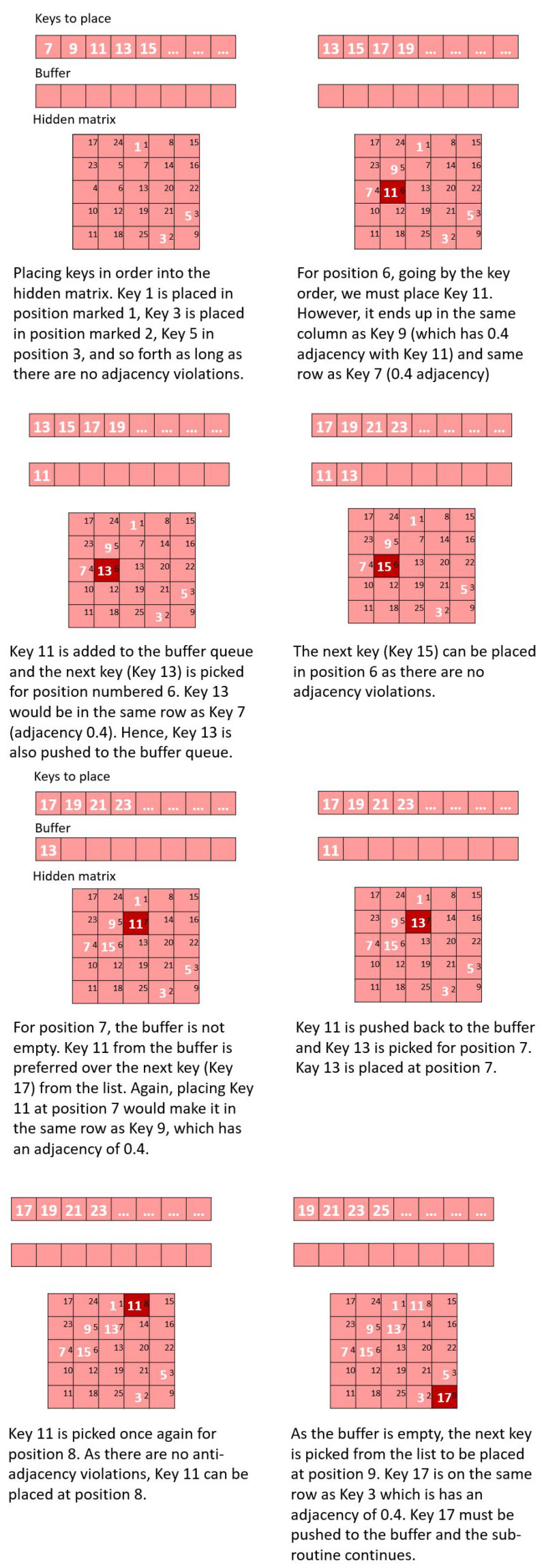
Anti-adjacency placement algorithm.

**Figure 14:**
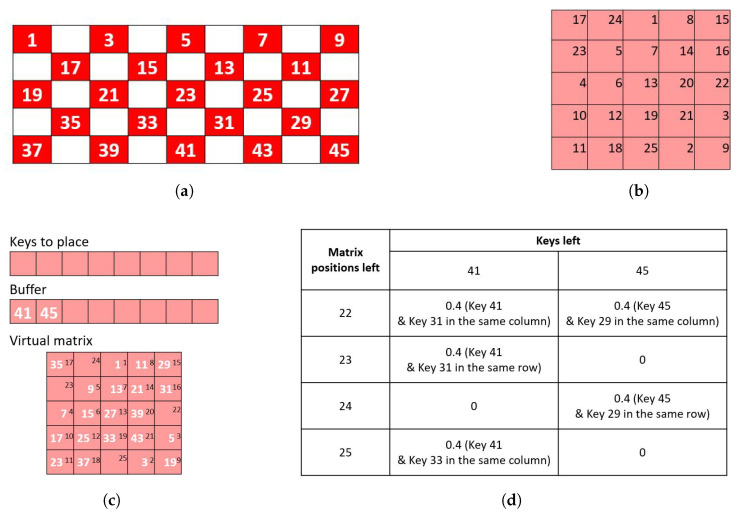
Illustration of steps to take if a situation is reached where none of the remaining keys (including those in the buffer queue) can be placed in the current position without two adjacent keys in the same row or the same column. (**a**) The 23 keys allotted to the red virtual matrix. The red virtual matrix is a 5 × 5 magic square. (**b**) The 5 × 5 magic square virtual matrix. (**c**) For the current position (location 22), all remaining keys cause some adjacency violation. (**d**) Adjacency calculation for all key–position pairs. The key–position pair that has the lowest adjacency is selected.

Our flash group creation algorithm is built on the CBP structure [27] with optimizations in four parameters:

- Virtual matrix dimensions—The dimensions of the two virtual matrices;
- Key order—The order in which keys are evaluated for placement in the virtual matrices;
- Board division—The method for dividing the keys between the two virtual matrices;
- Key placement—The method by which the keys are placed in the matrices;

For clarity and ease of reproducibility, we present the final form of the algorithm with the parameters that optimized performance. Appendix A describes the alternatives that were evaluated. We call the algorithm the *magic square paradigm (MSP)*, in reference to the selected key placement method. An overview of the algorithm is shown in Figure 7. Briefly, the steps of the algorithm are as follows:

1. Identify the location and dimensions of the keys to access.
2. Determine the size of square virtual matrices that will accommodate all the keys. Matrices should be square and approximately the same size (see Section 2.3.1).
3. Order the keys in each group for placement into the virtual matrices using switchback numbering (see Figure 8 and Section 2.3.2).
4. Divide the keys into 2 mutually exclusive groups using a checkerboard pattern (Figure 9). If the size of one of the groups exceeds the capacity of the largest virtual matrix, shift extra keys to the other group using the algorithm in Appendix B (see Figure 10 and Section 2.3.3).
5. Fill each virtual matrix according to a magic square pattern (Figure 11), using the anti-adjacency algorithm (Figure 12, Figure 13 and Figure 14) to minimize adjacency within the rows and columns of the matrix (see Section 2.3.4).

6. The rows and columns of the virtual matrices are the flash groups. Extract the row and column subgroups to create a flash group set in the order rows of matrix 1, rows of matrix 2, columns of matrix 1, columns of matrix 2.
7. Consecutive presentation of all flash groups constitutes a sequence. Between sequences, shuffle within the row and column subgroups to increase unpredictability.

#### 2.3.1. Virtual Matrix Dimensions

Building on the CBP [27], we use two virtual matrices to create flash groups. Since our algorithm accommodates variable numbers of keys, the size of the virtual matrices must change to match the number of active keys on the current AAC keyboard. While it is not necessary for the matrices to be square, square matrices support DP6: Equality, since the rows and columns of the virtual matrices are the flash groups. Square matrices also simplify the key placement algorithms (Section 2.3.4). We derive the size of the two virtual matrices from the number of active keys on the keyboard.

Given that the number of active keys on the keyboard is *k*, we search for a natural number *n* such that $2{\left(n-1\right)}^{2}<k\leq 2n^{2}$. Based on the value of k, we decide on the size of the virtual matrices.

- If
  $$2{\left(n-1\right)}^{2}<k\leq {\left(n-1\right)}^{2}+n^{2}$$
  the two virtual matrices are squares with sides (n−1) and *n*.
- Otherwise if
  $${\left(n-1\right)}^{2}+n^{2}<k\leq 2n^{2}$$
  the two virtual matrices are both squares with sides *n*.

We define the capacity of a virtual matrix as the maximum number of keys that can be accommodated in it ($n^{2}$ in the case of a square of side *n* and ${\left(n-1\right)}^{2}$ for a square of side (n−1)).

#### 2.3.2. Key Order

The *key order* parameter governs the assignment to each key of a number indicating the order in which it will be evaluated for placement into the virtual matrices (Section 2.3.4). Keys get their numbers based on their location on the grid, which is numbered in a consistent pattern. This is an important distinction. Each key will have the number for its location regardless of the number of active keys on the grid (Section 2.2: key-to-grid correspondence). An amalgamated key will be assigned the number of the top-left grid location it occupies. We tested four methods of assigning key orders: natural, switchback, diagonal, and diagonal switchback. Switchback was chosen and is described here. The other methods are described in Appendix A.

In the switchback grid number method, the top-left grid cell is assigned number 1. The numbers for each grid cell in the first row increase by one from left to right. After the first row is numbered, the rightmost cell on the second row is assigned the next number and the grid cells are then numbered from right to left. This pattern continues with odd-numbered rows numbered from left to right and even-numbered rows numbered from right to left, as illustrated in Figure 8. In keyboards with amalgamated keys, keys are numbered based the number of the leftmost and topmost cell they occupy on the grid (Figure 8c,d).

#### 2.3.3. Board Division

Three methods of dividing keys between the virtual matrices were evaluated: checkerboard, odd–even, and first-half/second-half. The checkerboard division was chosen and is described here. Odd–even and first-half/second-half are described in Appendix A.

For a fully populated AAC keyboard, the MSP checkerboard division into red and blue virtual matrices matches that of the CBP. Keys are assigned to the appropriate color matrix based on the color of their top-left grid location (Figure 9). A fully populated AAC keyboard with single cell keys divides the keys into sets of roughly equal sizes.

However, AAC keyboards with inactive locations or amalgamated keys may not divide into equal-sized sets. To account for such potential imbalances (e.g., Figure 10), the number of keys assigned to each matrix is compared against the size of the matrix. The larger set is always assigned to the larger matrix if the matrices are of different sizes. However, if one of the virtual matrices is allocated more keys than its capacity, keys are transferred from the over-capacity matrix to the alternate matrix until the imbalance is resolved.

A balancing sub-routine (Appendix B), implemented using a “greedy algorithm” approach, picks keys from the over-capacity matrix that are least adjacent to the keys in the complementary under-capacity matrix and transfers them sequentially until the over-capacity matrix has only enough keys to fill it completely. The adjacency of a key is calculated as the sum of adjacency with all keys in the complementary matrix. The keys with the lowest adjacency with the complementary matrix are transferred one by one to the new matrix. After transfer of one key, the adjacencies are recalculated. This prevents keys from the same region of the grid ending up in the same virtual matrix.

#### 2.3.4. Key Placement

The last step of the algorithm is to place the keys into the virtual matrices. The matrices are filled in a predetermined order. We evaluated three options for determining the placement order: magic squares, knight’s tour, and lead-diagonal. Magic square was chosen and is described here. The knight’s tour and lead-diagonal methods are described in Appendix A.

The placement order for the matrices is determined according to a magic square for that matrix size. Magic squares are a popular recreational mathematics tool. A magic square with sides of size m contains natural numbers from 1 through m^2^ such that all rows, all columns, and the major diagonals all sum to the same number, called the magic sum. The magic square placement numbering provides an underlying framework of a balanced distribution of keys and separates keys that are close together in key ordering.

Our algorithm uses magic squares with dimensions between 3 × 3 and 7 × 7. Only one magic square exists for a 3 × 3 square, but the number of possible magic squares grows rapidly with the size of the square. The number of size 6 magic squares is estimated to be on the order of $10^{20}$ [30]. We use the unique 3 × 3 square, along with arbitrarily selected magic squares of each size from 4 × 4 to 7 × 7, to provide the key ordering. The selected magic squares are shown in Figure 11.

Each matrix is filled with the keys allocated to it according to a greedy anti-adjacency algorithm that minimizes DP5: Anti-adjacency:

- Given a virtual matrix with numbered positions, the positions (in ascending order of the position numbers given by the magic square) are filled by keys (in ascending key order). So, among the keys allocated to the virtual matrix (by board division), the key that has the lowest key order is placed in the position numbered 1, the key with the second-lowest key order is placed in the position numbered 2, and so forth. Placement is subject to an anti-adjacency check to ensure that no two keys that have an adjacency metric greater than zero will be placed in the same row or column of the virtual matrix.
- If for a given virtual matrix position, the key that is about to be placed would violate the anti-adjacency directive (that is the current key would lie in the same row or column as another key whose adjacency with the current key is greater than zero), the key is placed in a buffer queue and the next key in order is picked.
- Once a virtual matrix position is filled, a key will be chosen for the next matrix position. If the buffer queue is not empty, the key is picked from the beginning of the buffer queue instead of the key order. The same rules of anti-adjacency apply to the key (the one picked from the buffer); if placing the key violates anti-adjacency, the key is pushed to the end of the buffer and a new key is picked (from the beginning of the buffer if the buffer is not empty or the next key in order if the buffer is empty). The steps so far are illustrated in Figure 12 and Figure 13.
- Eventually, there might come a point when it is not possible to place any of the remaining keys at the current virtual matrix position that prevents adjacency violations. In such a case, the adjacency violations are calculated for all remaining keys for all remaining virtual matrix positions. That is, if a key is placed in a position, the sum of the adjacency of the key with the other keys that have already been placed in the same row or column of the position are calculated. The key–virtual matrix position combination that has the lowest adjacency metric is chosen and the key is placed in that position. The adjacency violations are recalculated for the remaining keys and the remaining positions and the key–position pair that has the lowest adjacency violation is chosen. This final step of the sub-routine is illustrated in Figure 14.

The placement of keys using the key order, the magic square order and the greedy sub-routine is performed independently for the two virtual matrices. The rows and columns of the two virtual matrices are the final flash groups. A sequence is constructed by presenting the rows of the first matrix, followed by the rows of the second matrix, followed by the columns of the first matrix, followed by the columns of the second matrix. The order of presentation of each collection of rows and columns is varied for each sequence.

### 2.4. Parameter Optimization and Offline Analysis

As detailed in the previous sections, the algorithm has four parameters: virtual matrix dimensions, key order, board division, and key placement method. We tested many variants of these parameters and present in Appendix A the variants that produced promising results. These parameters were then exhaustively tested to confirm that the described combination of switchback key order, checkerboard board division, and greedy magic square key placement generates favorable flash groups for most keyboard configurations.

Parameter optimizations were initially done on a set of AAC keyboards (Appendix C). These keyboards were selected to represent the wide variety of keyboards and key arrangements. During optimization, the choice was made to concentrate on minimizing side adjacency and adjacency with amalgamated keys. It was noted early on that reducing side adjacency generally increased diagonal adjacency. Diagonal adjacency was considered undesirable, but of significantly less concern than side-adjacent keys that occupied the same row or column. So, parameter optimization decisions were based on the primary consideration of reducing side adjacency and adjacency with amalgamated keys. Diagonal adjacency was considered as a secondary factor.

To evaluate our algorithm, we tested it both on the 13 AAC screens used in the optimization process and on randomly generated keyboards where all keys occupy only a single cell in the grid. These keyboards are random in terms of which keys are active and which keys are ‘holes’. We created rectangular grids of sizes 4 × 7, 4 × 9, 5 × 9, 6 × 10, 7 × 12 and 9 × 16 and randomly filled 50%, 75%, or 100% of the grid locations. For each grid size and fill percentage combination, 25 random keyboards were generated. This produced 450 random keyboards (see Supplementary Materials).

The parameters board division, key order, and arrangement constitute the independent variables. There are 72 combinations of these parameters when considering all possible combinations of the 3 board divisions (‘firstsecond’, ‘oddeven’, and ‘checkerboard’), the 4 key order variants (‘natural’, ‘switchback’, ‘diagonal’, and ‘diagonalSwitchback’), and the 6 arrangement methods (‘knight’, ‘leaddiagonal’, ‘magic’, ‘greedy knight’, ‘greedy leaddiagonal’, and ‘greedy magic’).

Each of the 72 parameter combinations was tested up to 25 times on each of the 13 AAC screens and on each of the 450 random keyboards.In the 25 example cases, the locations for starting to fill the virtual matrices were varied in a controlled fashion. Thus, testing produced 23,400 test cases on AAC screens. However, testing on the random screens only produced 809,568 test cases. This was caused by one of the random keyboards for the 4 × 7 grid with the 50% fill rate only having 8 keys, so the magic square and lead diagonal placement options only had 16 combinations of starting locations in the two virtual matrices instead of the planned 25.

We evaluated the different parameter combinations on the six design principles. DPs 1 through 3 (identifiability, unpredictability, and perceptibility) are met by the underlying structure and thus not directly affected by the parameters. DP4, minimality, is fixed by the choice of virtual matrix dimensions. Thus the primary outcome metrics (dependent variables) were DP5 (anti-adjacency) and DP6 (equality). We calculated the percentage of flash groups that have the different types of adjacency (side, amalgamated, and diagonal). We also calculated the percentage of flash groups that contained any type of adjacency. We reported the mean across all the random keyboards (all sizes and fill percentages). We also calculated the largest difference in flash group size across all random keyboards. We implemented our algorithm in MATLAB R2023b (The MathWorks, Inc., Natick, MA, USA). MATLAB code is provided in the Supplementary Materials.

The parameter performance characteristics are presented sequentially; that is, all permutations of downstream parameters are used to evaluate the first parameter. At every step, the parameter is optimized based on the mean of the performance metric of all combinations of downstream parameters. Once the first choice has been made, the second parameter is evaluated using all permutations of the remaining downstream parameters. For example, if our choices are guided by DP5: Anti-adjacency, we look for sets of flash groups with the lowest fraction of groups that contain side-adjacent keys. Under such a prioritization objective, the ideal board division is where the mean number groups that contain adjacent keys is the lowest over all the choices for key order and key placement. In our experiment, we use our previously defined design priorities in that order: identifiability, unpredictability, perceptibility, minimality, anti-adjacency, and equality. The performance metrics for all parameter group combinations are provided in the Supplementary Materials so that other combinations can be examined if desired.

### 2.5. Implementation Considerations

We implemented MSP to create flash groups for access to the AAC keyboards of commercial AAC software from our industry partner. The Microsoft User Interface Automation (UIA) framework [31] was used to query the AAC software and obtain the sizes and locations of active keys. The MSP algorithm as specified here was used to create sets of flash groups for all keyboards with more than 8 keys. Flash groups with 8 or fewer keys were treated separately. The flash groups were used by the ERP-based BCI to overlay stimuli in a transparent window aligned on top of the AAC software. The UIA framework also enabled activation of specific keys, enabling access to the AAC software.

With 8 keys, virtual 2 × 2 matrices would be indicated. This would create 8 flash groups for the 8 keys and the minimum target-to-target interval would be 1 flash, reducing perceptibility. Indeed for any number of keys, *n* ≤ 8, the number of flash groups would be less than or equal to the number of keys. Thus, to maintain the design criteria of DP3: Perceptibility, *n* ≤ 8 keys should be treated differently.

Thus, we made the engineering decision that for *n* ≤ 8 keys, we create row and column flash groups for each key where the flash groups contain only that key. This creates 2n flash groups each containing one key. The rows are presented first and then the columns. Since rows and columns share the same keys, double flashes may occur when transitioning between row and column collections. If we encounter this during the sequence generation, we swap the head and tail of the group of rows or columns. These changes ensure DP3: Perceptibility. This approach was chosen over an explicit switch to single flashes because it did not require changes to the detection algorithm since each key is still in two groups.

Further, we implemented MSP with one addition to enhance DP2: Unpredictability and DP3: Perceptibility. As an algorithmic approach, the same set of flash groups would be created every time the algorithm was presented with the same set of keys. If a user struggles with a spatial arrangement of keys for any reason, it is desirable that different flash groups are presented in subsequent selections. To achieve this, our MSP implementation starts filling the magic squares from a randomly determined starting position each time. For example, in a 4 × 4 magic square, while the numbers start from 1 and go to 16, the first key can be placed at location 10 (instead of 1) and we can continue placing the next keys along the numbers. We cycle back to 1 when a key has been placed on location 16. By introducing this randomness, the algorithm ensures the flash groups themselves are random and unpredictable.

A final check was needed to handle a case that sometimes arose when the number of keys to place in a matrix was near the minimum for that matrix size. For example, if there were 10 keys to be placed in a 4 × 4 matrix, the filled matrix might include a completely empty row or column. This would create an atypical break in the flashes and a missing flash group number in the data that would complicate analysis. Thus, any empty rows or columns were removed (pruned) to further DP4: Minimality.

Computation time is a consideration for online implementation but was not considered critical since flashgroups were created during the setup for a new BCI selection, a period measured in seconds. The time required to create flash groups with the MATLAB implementation during optimization and testing was measured. Runtime was calculated for the 809,568 flash groups created for the random keyboards and the 19,800 AAC screens. Spot checking of the time required to create flash groups with the online C++ implementation was performed to identify potential problems.

### 2.6. On-Line Performance Analysis

The MSP flash group creation algorithm was implemented in C++ as part of a custom BCI2000 [32] application module. This implementation is in use in our current data collection studies. The AAC screens used in this study fell into 4 categories: the Unity keyboard (65 to 71 keys, depending on how many words were predicted), a simplified keyboard (49 to 55 keys depending on how many words were predicted), a pause screen (69 keys), and MINSPEAK^™^ keyboards (25 to 82 keys). We extracted the flash groups created during calibration and BCI use on these AAC screens and calculated the percentage of flash groups with adjacent keys and the maximum difference in size of the groups of keys.

## 3. Results

**Figure 15:**
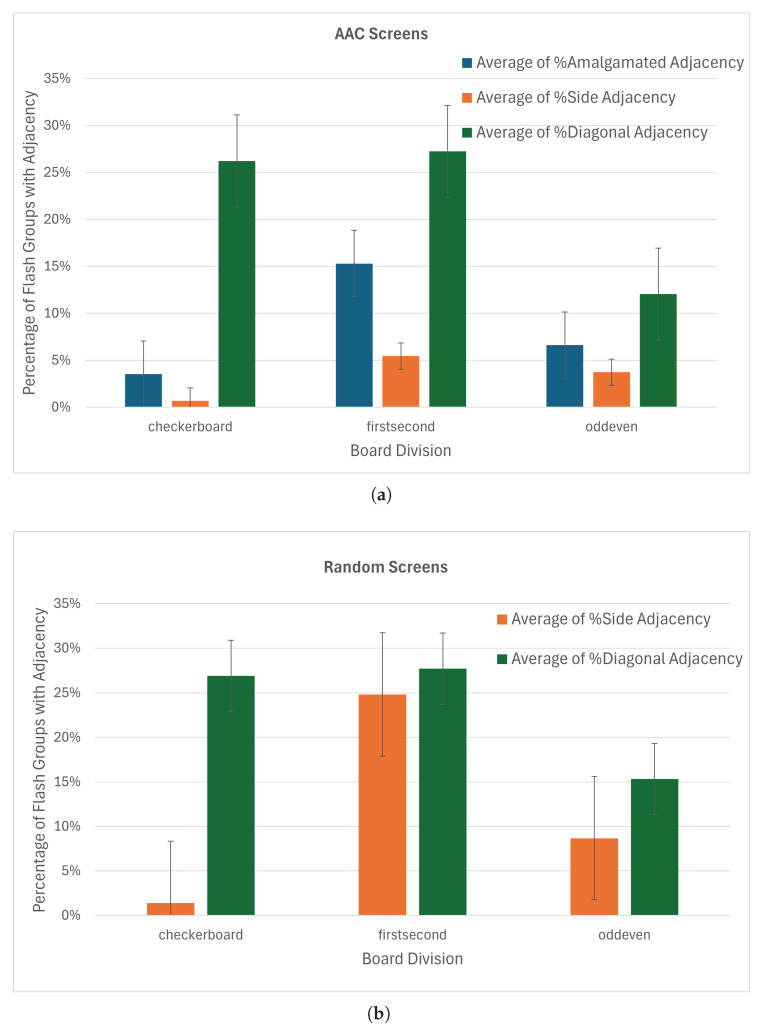
Percentage of flash groups that contained keys with different types of adjacency by board division. The error bars show the standard error. (**a**) AAC screens (columns represent average on 7800 flash groups). (**b**) Random screens (average on 269,856 flash groups).

The checkerboard split almost eliminates the incidence of side-by-side adjacent keys in the same flash group with an average of 0.7% of flash groups containing side-adjacent keys (Figure 15a). The incidence of adjacency with amalgamated keys is also less than for other board divisions, although diagonal adjacency is similar to first–second and greater than odd–even. The mean fraction of groups with adjacent keys for the checkerboard board division is less than that of the first-half/second-half and odd–even divisions. Hence, the choice of checkerboard for the board division parameter is clear.

**Figure 16:**
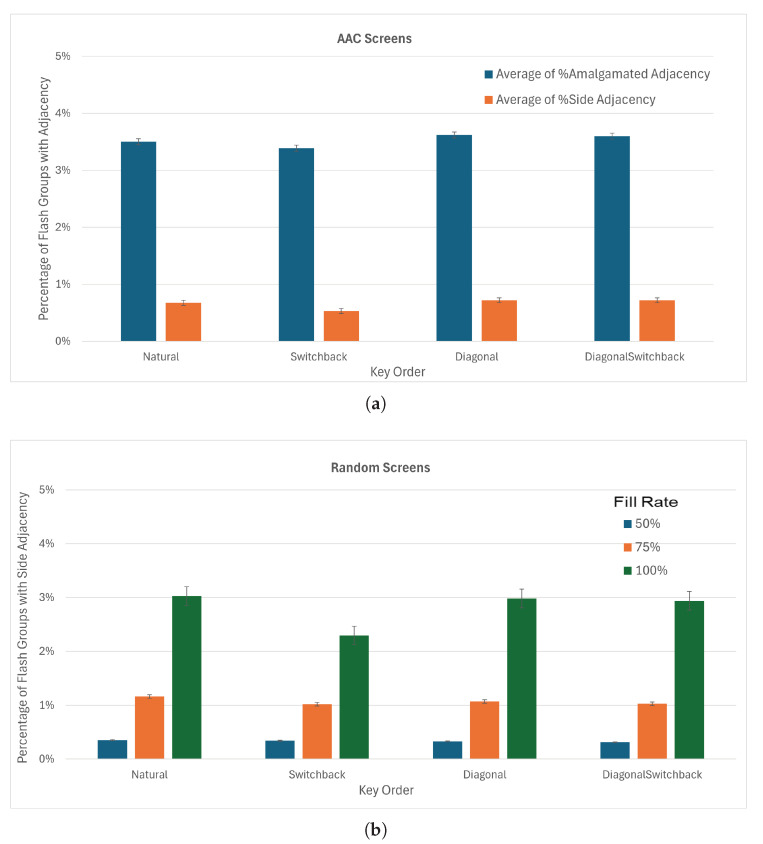
Percentage of flash groups that contained keys with different types of adjacency by key order after checkerboard board division. The error bars show the standard error. (**a**) AAC screens (columns represent average of 1950 flash groups). (**b**) Random screens (average of 22,464 or 22,500 flash groups).

Testing different key order options with the checkerboard board division did not show significant differences in adjacency (Figure 16b). The switchback ordering performed slightly better than natural ordering in amalgamated adjacency and side-by-side adjacency, as well as for fully populated random keyboards. With the checkerboard board division and the switchback key order, the average percentage of flash groups with side-adjacent keys dropped to 0.5% for the AAC screens and 1.2% for the random keyboards.

**Figure 17:**
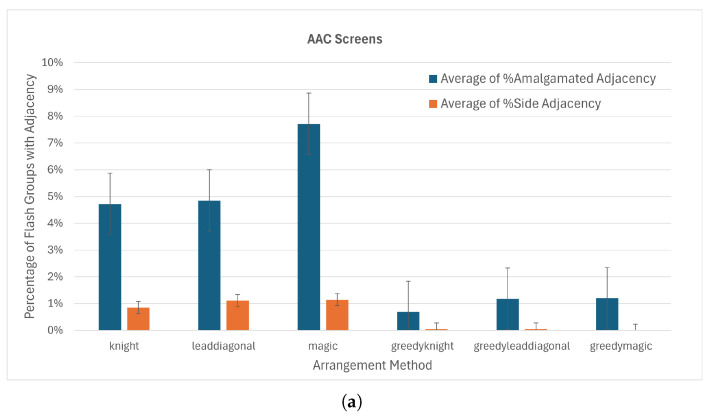

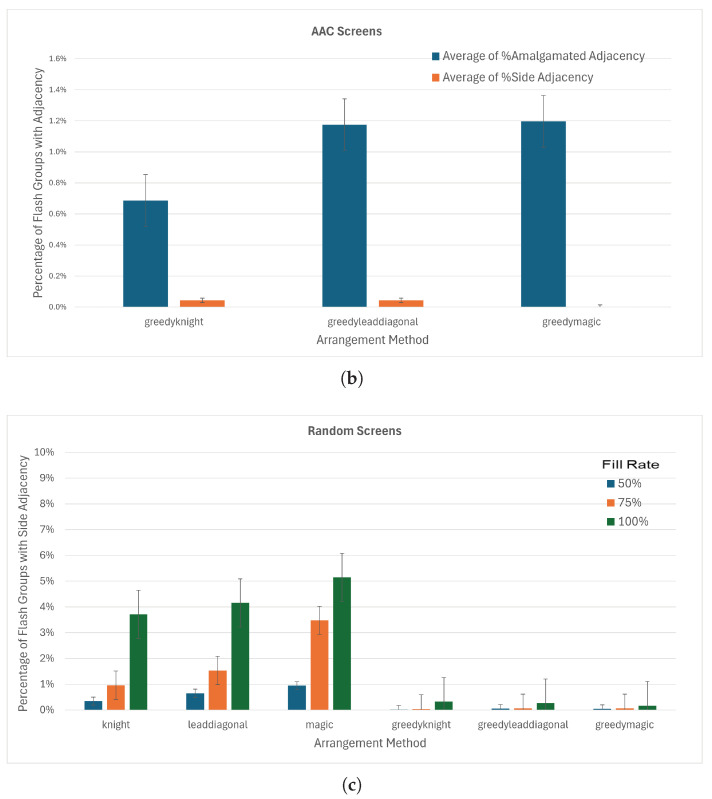
(**a**) Percentage of flash groups containing amalgamated-adjacent keys and with side-adjacent keys on AAC screens used for optimization (Columns represent average of 325 flash groups.). (**b**) Zoom on percentage of flash groups with adjacent keys in them by greedily placing keys. (**c**) Percentage of flash groups containing side-adjacent keys on random screens. (Columns represent averages on 3741 or 3750 flash groups).

All arrangement methods performed similarly on the AAC screens when using checkerboard board divisions and switchback key order, with side adjacency around 1% (Figure 17a). Using the greedy anti-adjacency algorithm (Section 2.3.4) decreased the side adjacency to 0 for the magic square arrangement method and amalgamated adjacency to 1.2% (Figure 17b). We therefore selected the magic square arrangement method for implementation online. When tested on the 450 random screens (Figure 17c), side adjacency was higher, especially for boards that were mostly populated, but remained under 1%. The knight’s tour placement appears to perform better for the random keyboards.

**Figure 18:**
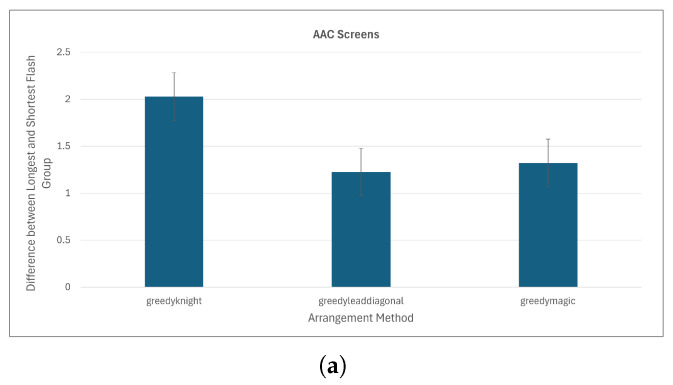

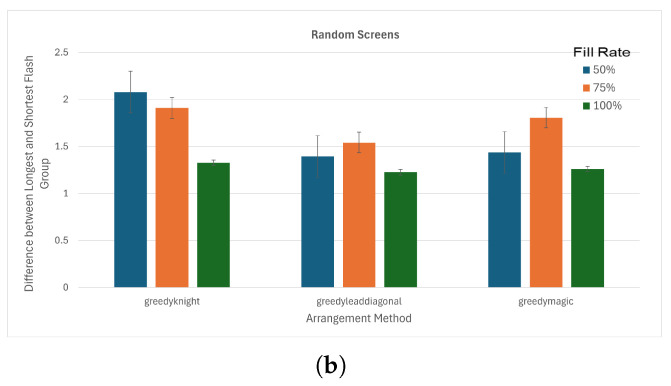
Average difference between the shortest and longest flash group in a set for (**a**) AAC Screens (columns represent the average of 325 flash groups), and (**b**) random keyboards (average of 3741 or 3750 flash groups. difference between sizes of flash groups—using checkerboard board division, switchback key ordering, and greedy placement.

The magic square and lead-diagonal placements (which calculate virtual matrix sizes in the same way) show the best performance in terms of equality of flash group sizes (Figure 18). With the greedy anti-adjacency algorithm on the AAC screens, magic square and lead-diagonal have average differences in flash group length of 1.3 and 1.2 keys respectively while knight’s tour has an average difference of 2.0 keys.

### On-Line Performance

Data was available from MSP use for access to AAC screens for 5778 selections (see details in Table 1). Unity and MINSPEAK^™^ keyboards were only used during BCI calibration and thus have fewer selections and a consistent set of MINSPEAK^™^ screens on seven unique screens. There were 126,944 flash groups created for the 5778 selections. There were no occurrences of side-by-side adjacency between single-cell keys. There were 20 occurrences (0.02%) of adjacency that included overlap with amalgamated keys. Diagonal adjacency between keys occurred in 6% of groups. The average difference between the shortest and longest flash groups was 1.9 keys.

**Figure 19:**
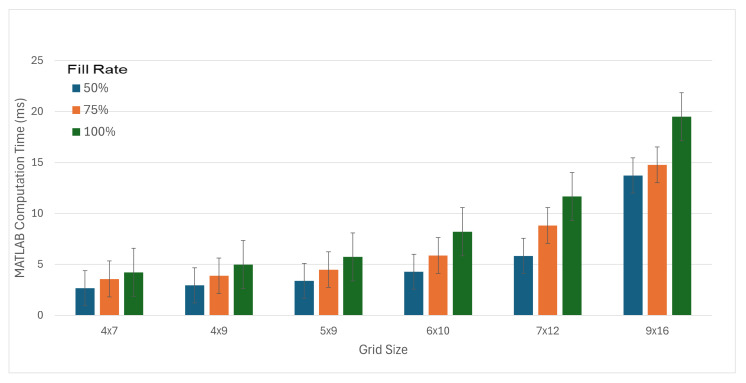
Average runtime for MATLAB implementation of MSP flash group creation for all parameter combinations by board size and fill rates. Each bar represents the average of 44,568 or 45,000 flash group computations. Error bars show standard error.

The time required to create flash groups was measured during the simulation for testing on random keyboards. Computation time required an average of 7.1 ms with a standard deviation of 5.4 ms. Figure 19 shows the computation time for different board sizes and fill rates. In the online C++ implementation, computation time (as reported by timestamps with ms precision) never took more than 1 ms.

## 4. Discussion

Section 4.1 discusses the effectiveness of the MSP algorithm in satisfying the design priorities. Section 4.3 discusses specific vulnerabilities in the MSP algorithm and the results produced. Section 4.4 discusses the limitations of this study. Section 4.5 draws conclusions and describes future directions.

### 4.1. Evaluation of Design Priorities

Table 2 shows the relationship between design priorities, design mechanisms, and design evaluation metrics. We discuss each in turn.

The first three design priorities (DP1: Identifiability, DP2: Unpredictability, and DP3: Perceptibility) are guaranteed by the structure of the virtual matrices as originated in the CBP.

DP1: Identifiability is guaranteed through the paradigm of selecting keys at the intersection of two groups. By placing all keys in exactly two flash groups, all keys can be theoretically ascertained from exactly one flash of each flash group. This essential requirement of DP1: Identifiability is also satisfied by the RCP and the CBP.

DP2: Unpredictability is guaranteed by the use of virtual matrices that break the link between spatial locations and flash groups. This is further enhanced by shuffling the rows and columns of each matrix between sequences and by filling the magic squares from a different point each time the algorithm is used.

Unpredictability can be quantified as the variance of TTI. TTI = 0 when consecutive flashes contain the same key. The constraints imposed by DP3 tell us that, for matrices of size *n*, TTI ranges from n−1 to 3n−2. Because each subsequence (e.g., row 1, row 2, column 1, column 2) is randomized, each TTI value between n−1 and 3n−2 is equally likely to occur. A discrete uniform distribution over a population of size *N* has variance $(N^{2}-1)/12$; since N=(3n−2)−(n−1)+2n, the variance of TTI in terms of n is $({\left(2n\right)}^{2}-1)/12$.

DP3: Perceptibility is guaranteed by how the flash groups are presented (Figure 7). All rows (or columns) of the first virtual matrix are presented before moving on to the rows (or columns) of the second matrix. Thus, the minimum number of flashes before any key repeats is equal to the size of the smaller matrix. As the size of matrices grows, the minimum duration between two consecutive flashes of the same key also increases. Thus, the important characteristic TTI [28] is maximized.

Minimum and maximum TTI can be calculated based on the dimensions of the hidden matrices. For two n×n matrices, TTI ranges from *n* to 3n−2. For an n×n and an (n−1)×(n−1) matrix, TTI ranges from n−1 to 3n−3.

DP4: Minimality is facilitated by choosing the size of virtual matrices so that there are just enough positions (the capacity of the virtual matrices) to place all keys while maintaining the square shape of the virtual matrices. This is further enhanced by the pruning of any empty rows or columns.

DP5: Anti-Adjacency is facilitated by the sub-routine that balances the keys after an imbalanced board division and the magic square key placement with the greedy anti-adjacency sub-routine. When violations are inevitable, the algorithm attempts to reduce the impact of such violations by using the adjacency metric.

DP6: Equality is facilitated by the square virtual matrices. While the variable numbers of keys create situations were the key numbers are not evenly divisible, square matrices minimize the number of such instances.

### 4.2. Practical Considerations

Our use of MSP flash group creation was performed in the time between BCI selections: that is, after the BCI had made a selection and while it was aligning to a potentially new screen layout. This time is measured in seconds since it is also the time during which the user is observing the selection made by the BCI and deciding on their next target key. Implementation efficiency was therefore not a concern during implementation. However, the observed MSP runtimes show that flash groups could be recalculated after every sequence. Our ERP BCI analyzes 800 ms of brain activity after each stimulus and stimuli are presented for 90–150 ms with gaps of 30–60 ms between flashes. So, a sequence of 22 flash groups takes 3960 ms. Thus, the 7.1 ms required for MATLAB to calculate a new set of flashes, or even the 16.0 ms for the 9 × 16 grid, is sufficiently small so as not to be a concern. Algorithm complexity for numbers of keys larger than those tested is not a concern since the 144-key maximum used on the 9 × 16 AAC screen far exceeds the number of clickable objects present on everyday software.

### 4.3. Algorithm Vulnerabilities

MSP flash group creation is now in regular use in our studies. Improvements in the flash patterns were perceptible with both the addition of magic square placements and the greedy placement algorithm. Nevertheless, occasional odd flash groups were observed. Analysis of the flash groups generated during online use highlights specific conditions that exploit vulnerabilities in the MSP, particularly regarding DP5: Anti-Adjacency and DP6: Equality.

DP6: Equality can be violated when the number of keys only slightly exceeds the capacity of the selected hidden matrices. For example, our simplified keyboard has 49 keys when word prediction is not active and 55 keys when all six word prediction keys are active. However, key numbers in the range of 49 to 55 are also possible if there are few word prediction possibilities. A keyboard with 51 keys will have one 6 × 6 matrix and one 5 × 5 matrix (capacities 36 and 25). The greedy placement algorithm minimizes adjacency but does not evaluate equality of flash group size. Analysis of online performance revealed six sets of flash groups (out of 5778 examples) where DP6: Equality was violated because flash group sizes ranged from one to six keys. This occurred for keyboards with 51, 52, or 53 keys. An additional 44 flash group sets had flash group lengths from two to six keys, causing a length difference of four keys. These cases are rare (0.1% and 0.8% respectively), but they can be distracting when they occur.

Another vulnerability is adjacency between amalgamated keys. The vulnerability to amalgamated keys forcing imbalanced board divisions is described in Section 2.3.3 and illustrated in Figure 9. The location of the space bar at the bottom of the keyboard (giving it a late placement number) probably increases the likelihood of it being forced into a placement that creates adjacency. A single-cell key that is placed next to the long side of an amalgamated key will have an adjacency value of 1.4 or greater since it shares both a side (adjacency of 1) and a diagonal (adjacency of 0.4) with the amalgamated key. This situation occurred in 20 out of 5778 (0.3%) flash group sets in our online test data. While reversing the order of key placement on the screen might minimize adjacency for the space bar, the amalgamated word prediction keys near the top of the screen would then be vulnerable. A better approach might be to put all amalgamated keys early in the key order. However, predicting how this approach will interact with the other parameters is difficult. Benefit might also be gained by intentionally equalizing the visual impact of flashes, not just by designing for equal-sized flash groups but by intentionally balancing the surface area of the keys in a flash group. Further study is needed.

The MSP parameters do not appear to be unique when tested on random keyboards. The knight’s tour choices also show promise, though they increase the total number of flash groups, which would increase the time for one selection with the BCI.

### 4.4. Limitations

Several limitations should be acknowledged. First, the flash group creation algorithm was designed to access a specific set of on-screen keyboards and tested for alignment to the stated design criteria. Testing the information transfer rate or accuracy differences during online BCI use between MSP flash groups and RCP or CBP flash groups was not considered practical because the application of RCP and CBP to partly populated keyboards is undefined. Second, MSP flash groups were only tested on rectangular keys presented in a known grid structure. Finally, adjacency weightings were empirically determined and not fully optimized. The current adjacency weights of 1.0 for side adjacency and 0.4 for diagonal adjacency give preference to a side-adjacent placement over a placement that causes three diagonal adjacencies.

### 4.5. Conclusions and Future Work

The MSP algorithm effectively addresses the six design priorities for creating flash groups to support an effective ERP-BCI. The MSP algorithm can create flash groups from any distribution of rectangular keys on a grid, including keyboards containing multiple key sizes and inactive key locations.

Future work should include addressing identified vulnerabilities, especially those resulting from keys that combine two grid squares, forcing adjacent keys into the same color matrix. This work could be combined with work to derive a compatible grid structure from the size and location of keys for programs without a pre-defined underlying grid structure. Another beneficial optimization would define additional rules for the placement of amalgamated keys, perhaps by prioritizing their placement in the hidden matrices. A final addition would be to define adjacency rules for non-rectangular keys, enabling flash groups to be created to access any arbitrary keyboard.

The current MSP algorithm makes BCI access possible for many commercial AAC devices and simple programs. Additional work should support expanding that access and making many existing programs accessible with BCI. 

## Figures and Tables

**Table 1:** Adjacency results by screen type for online BCI use.

Keyboard	Number of Selections	Number of Groups	Groups with Adjacency	Groups with Amalgamated Adjacency	Groups with Side-Adjacency	Groups with Diagonal Adjacency	Difference in Flash Group Length
Unity	127	2744	91	0	0	91	1.3
UnityKey	93	2236	128	15	0	113	1.3
PauseScr	1040	24,960	840	0	0	840	1.3
keyboard	4518	97,004	7083	5	0	7078	2.1
Total	5778	126,944	8142	20	0	8122	1.9

**Table 2:** Mapping of design priorities to design mechanisms and evaluation metrics.

Design Priority	Design Mechanism	Evaluation Metric
DP1: Identifiability	Hidden matrices	Each key associated with unique set of flash groups
DP2: Unpredictability	Shuffle of flash groups between sequences	Variance of target-to-target interval
DP3: Perceptibility	Ordering of groups by rows and columns of hidden matrices	Average target-to-target interval
DP4: Minimality	Different-sized hidden matrices; pruning empty rules and columns	Number of flash groups
DP5: Anti-adjacency	Checkerboard division; magic square placement; anti-adjacency placement algorithm	Adjacency measures; metrics for amalgamated adjacency, side adjacency, and diagonal adjacency
DP6: Equality	Magic square placement distributed over the hidden matrix	Difference between minimum and maximum length

**Figure A1:**
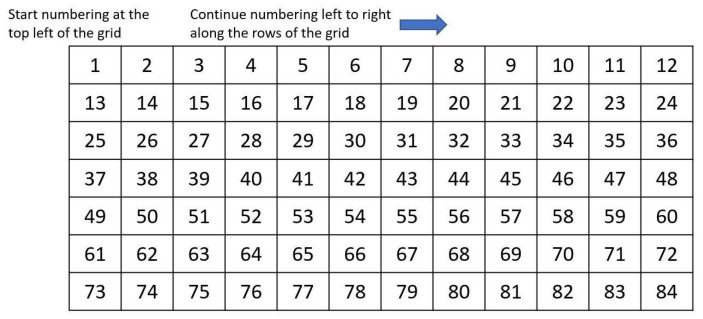
Natural grid cell ordering.

**Figure A2:**
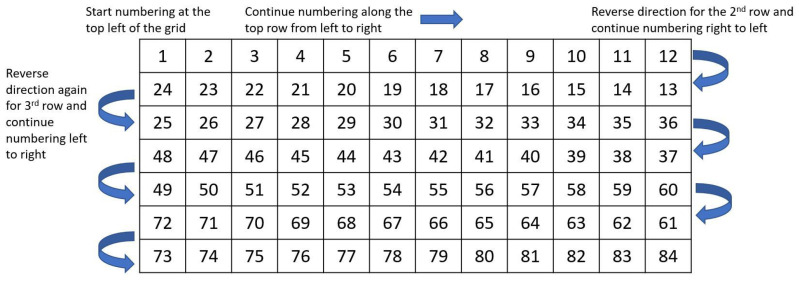
Switchback grid cell ordering.

**Figure A3:**
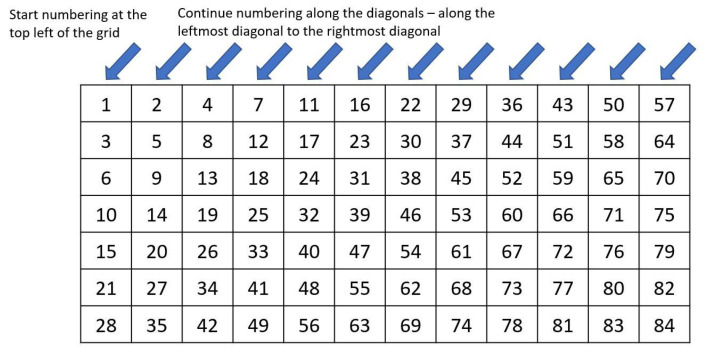
Diagonal grid cell ordering.

**Figure A4:**
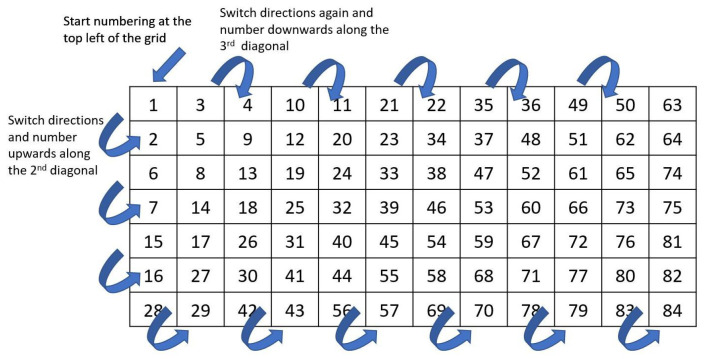
Diagonal switchback grid cell ordering.

**Figure A5:**
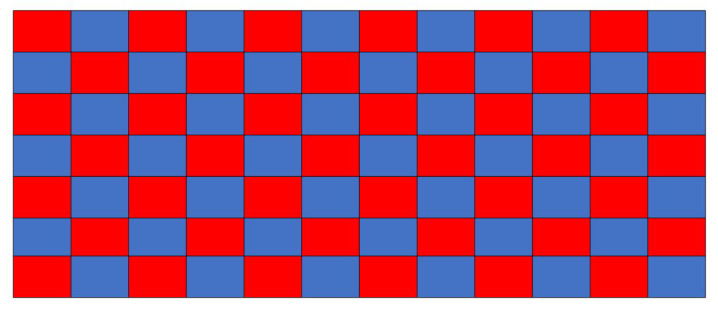
Checkerboard division. The cells are split according to a checkerboard pattern.

**Figure A6:**
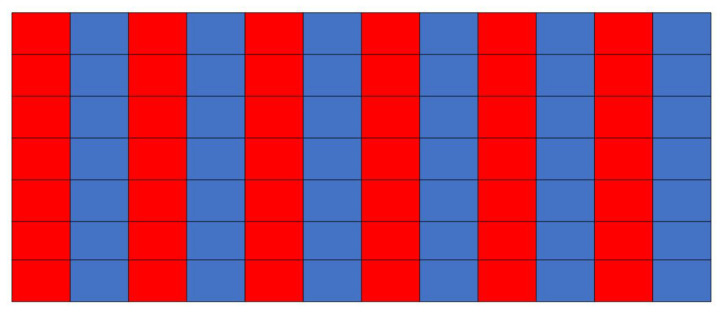
Odd–even division. Every odd key is in the first matrix and every even key is in the second matrix. In the case of grids with an odd number of columns, the odd–even and the checkerboard split are equivalent.

**Figure A7:**
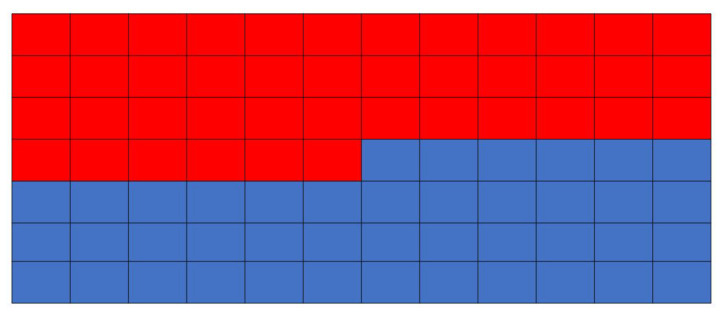
First-half/second-half division. Keys in the first half of the grid are in the first matrix and keys in the second half of the grid are in the second matrix.

**Figure A8:**
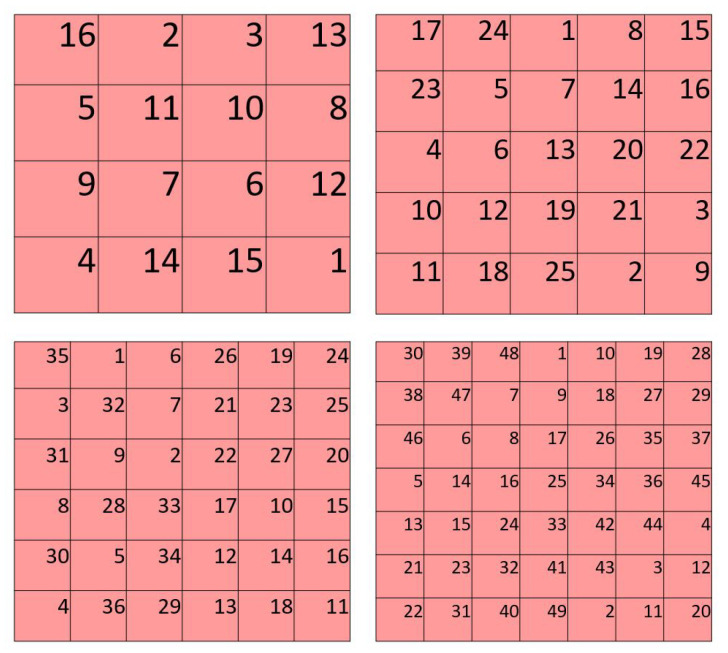
Magic squares. Numbering is based on magic squares where the sum of numbers along all the rows, the columns and the two diagonals are the same.

**Figure A9:**
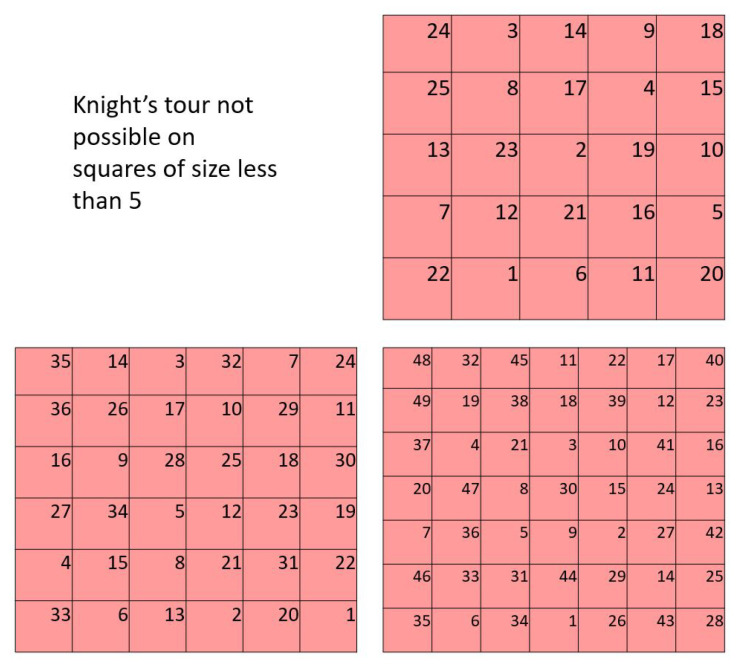
Knight’s tour. Numbering is based on the sequence in which a chess knight moves on a square grid such that it visits each cell exactly once. Since knight’s tours are not possible on squares of size less than 5, for key numbers less than 25, rectangular matrices were used [33].

**Figure A10:**
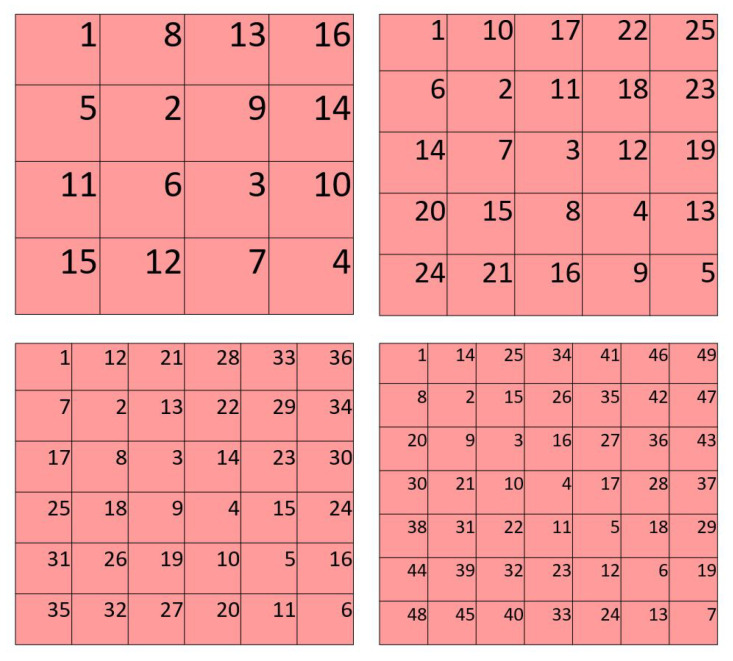
Lead-diagonal. Numbering proceeds along the lead-diagonal and then alternates on either side of the lead-diagonal.

**Figure A11:**
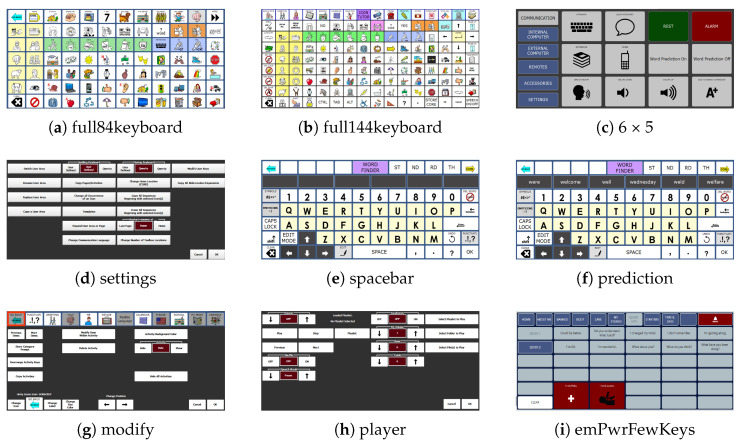

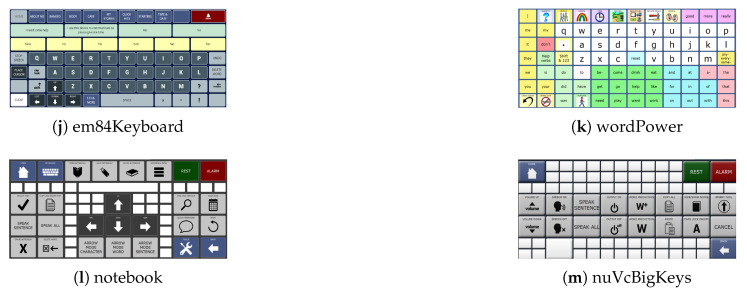
The AAC keyboards used for parameter optimization with the names to load their specifications from LayoutSample.m in the Supplementary Materials.

## Data Availability

All relevant data is contained within the article.

## Supplementary Materials

The following supporting information can be downloaded at https://www.mdpi.com/article/10.3390/s26072123/s1. File AACScreensControlledStart.csv: The data from flash group examples on the AAC Screens; File OnlineFlashGroups.csv: The data from the on-line flash group creation; File RandomScreensControlledStart.csv: The data from flash group examples on the random keyboards; File MATLAB-Files.zip: The MATLAB code to create flash groups and run the comparisons. Includes scripts topLevelExample.m to create and view a small set of flash groups, Test_methods_on_AAC_Keyboards.m to run examples on all AAC_Keyboards, Test_methods_on_Random_Keyboards.m to run examples on random keyboards, Random_Keyboards.mat, the random keyboards used, and the 17 MATLAB function files needed by the scripts.

## Abbreviations

The following abbreviations are used in this manuscript:

AAC	Alternative and augmentative communication
BCI	Brain–computer interface
CBP	Checkerboard paradigm for flash group creation
DP	Design priority
EEG	Electroencephalogram
ERP	Event-related potential
ms	Milliseconds
MSP	Magic square paradigm for flash group creation
NIDCD	National Institute of Deafness and Other Communication Disorders
NIH	National Institutes of Health (United States)
RCP	Row–column paradigm for flash group creation
TTI	Target-to-target interval
UIA	User interface automation

## Appendix A. Alternate Algorithm Parameters

To arrive at the MSP design presented in Section 2: Methods, we tested several options for key order, board division, and key placement. Here we present all the alternatives that were considered.

### Appendix A.1. Alternate Key Orders

The ordering of the keys is based on how the cells of the underlying rectangular grid are numbered. We tested four variants: natural (Figure A1), switchback (Figure A2), diagonal (Figure A3), and diagonal switchback (Figure A4).

### Appendix A.2. Alternate Board Divisions

Similarly to the key order, the division of the keys into red and blue virtual matrices is dependent on which grid cells the keys occupy. We tested three variants: checkerboard (Figure A5), odd–even (Figure A6), and first-half/second-half (Figure A7).

### Appendix A.3. Alternate Key Placements

Keys are placed into the virtual matrices based on a predetermined order of placement. Three options for those numberings were evaluated: magic square (Figure A8), knight’s tour (Figure A9), and lead-diagonal (Figure A10).

## Appendix B. Virtual Matrix Capacity Shuffle Sub-Routine

In cases where the checkerboard pattern creates two divisions such that one of the virtual matrices is assigned keys numbering more than its capacity, keys must be moved from the over-capacity matrix into the other matrix. The keys to move are identified by the following steps:

- For all keys in the over-capacity virtual matrix, calculate the sum of adjacencies with all keys in the other matrix.
- If the matrix is over-capacity by $e_{k}$, pick the $e_{k}$ keys with the lowest adjacencies with the other matrix. Let us call this set the tentatives and let the maximum adjacency with the other matrix of the tentatives set be $adj_{max}$.
- Calculate the within-adjacency of each key in the tentatives with every other key in the tentatives. Calculate the total adjacency of every key in the tentatives as the sum of the cross-adjacency (with the other matrix) and the within-adjacency (within the tentatives set).
- Consider all remaining keys in the over-capacity matrix whose adjacency with the other matrix is less than or equal to $adj_{max}$. Let us call this set probables.
- Pick a key from the tentatives and swap it out with a key from probables. Calculate the within-adjacencies of the new key. If the total adjacency of the new key is less than the total adjacency of the key that was swapped out, retain the new member as a permanent member.
- Repeat the above step for all members of the tentatives set.
